# 27337 Characterizing Temporal Patterns in Glucose Dysregulation Following SARS-CoV-2 Infection

**DOI:** 10.1017/cts.2021.523

**Published:** 2021-03-30

**Authors:** Sejal Mistry, Ramkiran Gouripeddi, Julio C. Facelli

**Affiliations:** Department of Biomedical Informatics University of Utah

## Abstract

ABSTRACT IMPACT: Understanding the longitudinal glucose changes following SARS-CoV-2 infection can inform point-of-care guidelines and elucidate the viral hypothesis of diabetes mellitus pathogenesis. OBJECTIVES/GOALS: Hyperglycemia has emerged as an important manifestation of SARS-CoV-2 infection in both diabetic and non-diabetic patients. Whether clinically-detectable glycemic changes persist following SARS-CoV-2 infection remain to be elucidated. This work aims to characterize temporal patterns in glucose dysregulation following SARS-CoV-2 infection. METHODS/STUDY POPULATION: Electronic health records of patients with a diagnosis of COVID-19, positive laboratory test for SARS-CoV-2, and negative history of Diabetes Mellitus prior to infection were extracted from the TriNetX database. 7,502 patients with at least one blood glucose value 2 years to 2 weeks before, 2 weeks before to 2 weeks after, and 2 weeks after to 1 year after COVID-19 diagnosis were used for analysis. Temporal patterns are characterized by training state-of-the-art clustering algorithms, including fuzzy short time-series clustering, k-means for longitudinal data, and spectral clustering. Clustering performance is evaluated using internal evaluation metrics of the Silhouette coefficient, Calinski-Harabasz score, and Davies Bouldin index. RESULTS/ANTICIPATED RESULTS: Based on the success of prior clustering methods with random blood glucose measurements, we anticipate that the proposed time-series clustering algorithms will appropriately characterize temporal patterns of glycemic dysregulation. The best performing algorithm based on interval evaluation metrics will be selected for further analysis. Associations between blood glucose values and cluster membership will be evaluated using Kruskal-Wallis one-way ANOVA and effect size will be calculated using unbiased Cohen’s d. Clinical phenotypes for each cluster will be characterized in terms of current diagnoses, prior medication use, pertinent laboratory tests, and vital signs. DISCUSSION/SIGNIFICANCE OF FINDINGS: A clearer understanding of the longitudinal glucose changes following SARS-CoV-2 infection can elucidate clinically-detectable patterns of glycemic dysregulation, identify sub-phenotypes of patients who are more susceptive to glycemic dysregulation, and inform appropriate point-of-care guidelines.

